# Recognition of Noisy Radar Emitter Signals Using a One-Dimensional Deep Residual Shrinkage Network

**DOI:** 10.3390/s21237973

**Published:** 2021-11-29

**Authors:** Shengli Zhang, Jifei Pan, Zhenzhong Han, Linqing Guo

**Affiliations:** Electronic Countermeasure Institute, National University of Defense Technology, Hefei 230037, China; zhangshli@mail2.sysu.edu.cn (S.Z.); hzz@nudu.edu.cn (Z.H.); guolq@nudt.edu.cn (L.G.)

**Keywords:** radar emitter signal recognition, high noise, one-dimensional residual shrinkage network, soft thresholding

## Abstract

Signal features can be obscured in noisy environments, resulting in low accuracy of radar emitter signal recognition based on traditional methods. To improve the ability of learning features from noisy signals, a new radar emitter signal recognition method based on one-dimensional (1D) deep residual shrinkage network (DRSN) is proposed, which offers the following advantages: (i) Unimportant features are eliminated using the soft thresholding function, and the thresholds are automatically set based on the attention mechanism; (ii) without any professional knowledge of signal processing or dimension conversion of data, the 1D DRSN can automatically learn the features characterizing the signal directly from the 1D data and achieve a high recognition rate for noisy signals. The effectiveness of the 1D DRSN was experimentally verified under different types of noise. In addition, comparison with other deep learning methods revealed the superior performance of the DRSN. Last, the mechanism of eliminating redundant features using the soft thresholding function was analyzed.

## 1. Introduction

One of the most important functions of radar countermeasure systems is that radar emitter signal recognition, in which classification and recognition of intercepted radar signals are carried out to determine the radar type, purpose, carrier, threat level, and recognition credibility of the radar [1]. Therefore, accurate radar emitter signal recognition is essential for subsequent radar analysis and action preparation.

The existing radar emitter signal recognition methods can be divided into two categories. The first category includes traditional signal analysis methods, including gray correlation analysis [2], template matching [3], fuzzy matching [4], and attribute measurement [5]. However, there are various deficiencies in the traditional methods, the recognition performance is dependent on the richness of prior knowledge, tolerance rate and robustness are poor, and they do not have automatic learning abilities. The second category includes deep learning methods, which are usually based on time-frequency transform. For example, the classification and recognition of signals were achieved using the Choi–Williams time-frequency distribution with convolutional neural network (CNN) [6]. In a study by Zhao et al., the Margenau–Hill time-frequency distribution and smooth pseudo-Wigner–Ville distribution (SPWVD) were used as signal features, and then a classifier was built for radar emitter signal recognition based on an automatic encoder (AE), a deep belief network (DBN), and a CNN [7]. Based on the deep Q-learning network (DQN) [8], the Cohen’s class time-frequency distributions were used for signal recognition. Wu et al. [9] used one-dimensional (1D) CNN for radar signal recognition. However, the parameter optimization of traditional deep learning methods is a difficult task, the error function gradient may gradually become inaccurate in the process of reverse propagation. When the network layer is too deep, the parameters in the initial layers cannot be optimized well. Moreover, the 1D raw signal needs to be converted to a two-dimensional signal, consuming extra time and calculation resources.

With the development of radar technology, the electromagnetic environment is becoming increasingly complex, and received radar signals are inevitably accompanied by noise [10,11]. What is more tricky is that different types of noise have different impacts on radar signals. Traditional signal analysis and processing methods are effective only for a certain type of noise. Besides, the recognition accuracy is low under strong noise conditions. For deep learning methods, the learned high-level features are likely to be less discriminative under the interference of noise. Therefore, it is necessary to develop a new radar emitter signal recognition model that not only has the ability to process different types of noise, but can also achieve a high recognition rate under strong noise conditions. To achieve the above requirements, in this study, a radar emitter signal recognition method based on a 1D deep residual shrinkage network (DRSN) is proposed.

The main contribution of this study are as follows:

(1) Important features could be directly extracted from a time-sequential sequence using a 1D DRSN without dimension conversion. Compared with traditional deep learning methods, the recognition accuracy was improved.
(2) The rectified linear unit (ReLU) was replaced by a soft thresholding function to eliminate unimportant features. Moreover, the attention mechanism was used to adaptively set the threshold to achieve recognition of noisy radar emitter signals. The mechanism of elimination of redundant features using the soft thresholding function was analyzed.
(3) Radar emitter signals containing different types of noise were recognized using the proposed method, showing excellent results.

The rest of this article is organized as follows. Four noise models are proposed in Section 2, and the structure of the 1D DRSN is proposed in Section 3. Section 4 presents tests and data analysis, followed by the elucidation of the denoising mechanism of the soft thresholding function in Section 5. Finally, the study is concluded in Section 6.

## 2. Signal Noise

We do not know what types of noise it is when intercepting a radar emitter signal. Thus, the ability to process different types of noise is essential for a radar emitter signal recognition model. The following four representative types of noise were used as the background noise of the radar emitter signal, and then the 1D DRSN was used for recognition.

### 2.1. Gaussian Noise

With strong randomness, gaussian noise widely exists in the environment [12]. Under a low signal-to-noise ratio (SNR), Gaussian noise can severely impact the time-domain waveform of the signal and cover up useful information, resulting in difficulty in signal recognition. As a type of Gaussian noise, white Gaussian noise is often added to a signal to form additive white Gaussian noise in communication channels for testing and modeling, where the probability density function is as follows:

(1) $$f\left(x\right)=\frac{1}{\sqrt{2\pi }\sigma }e^{-{\left(x-\mu \right)}^{2}/2\sigma^{2}}$$

where x is a random variable, and μ, σ are the mean and standard deviation of the Gaussian distribution, respectively.

### 2.2. Laplacian Noise

Laplacian noise is a non-Gaussian noise. There are often pulse noise and co-channel interference in the actual communication environment [13], causing the Gaussian noise model to no longer be applicable, so it is essential to recognize radar emitter signals with non-Gaussian noise. In this study, Laplacian noise was used as a non-Gaussian noise for testing the performance of the proposed model. The Laplacian probability density function is as follows:

(2) $$f\left(x\right)=\frac{1}{2\lambda }e^{\frac{-\left|x-\mu \right|}{\lambda }}$$

where x is a random variable and λ, μ are constants.

### 2.3. Poisson Noise

Poisson noise is a signal-dependent noise. Different from the distribution of white Gaussian noise, which is independent of the signal, the distribution of Poisson noise is closely related to the signal; i.e., there is a strong correlation between the noise intensity and signal intensity [14]. The traditional methods for processing additive Gaussian noise are not applicable to Poisson noise. Considering the advantage of deep learning-based methods with self-learning discriminant features, in this study, radar emitter signals with Poisson noise were recognized using the 1D DRSN method. The probability density function of Poisson noise is as follows:

(3) $$f\left(k;\lambda \right)=\frac{\lambda^{k}e^{-\lambda }}{k!}\;\;\left(k=0,1,\cdots \right)$$

where λ is the average frequency of a random event per unit time.

### 2.4. Cauchy Noise

Cauchy noise is a kind of Lévy noise, which has a heavy-tailed probability distribution [15]. In the field of radar emitter signal recognition, Cauchy distributions can be used to simulate burr noise environment. The probability density function of Cauchy noise is as follows:

(4) $$f\left(x;\mu ;\sigma \right)=\frac{1}{\pi }\left[\frac{\sigma }{{\left(x-\mu \right)}^{2}+\sigma^{2}}\right]$$

where μ is the location parameter, and σ is the scale parameter.

## 3. One-Dimensional Deep Residual Shrinkage Network (1D DRSN)

### 3.1. 1D Convolution

**Figure 1:**
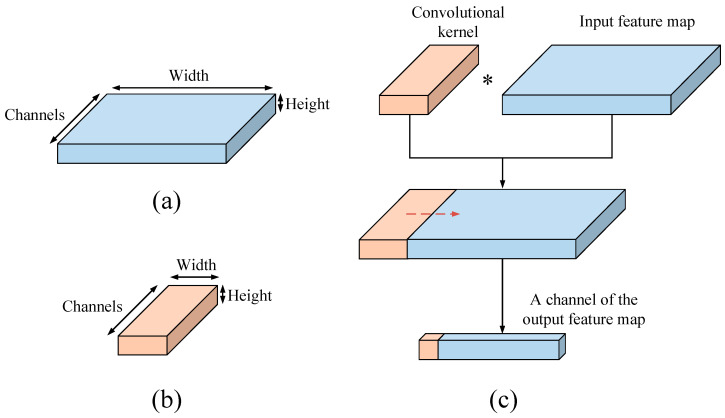
One-dimensional convolution, (**a**) input features, (**b**) convolutional kernel, and (**c**) the 1D convolution process.

In the proposed 1D DRSN method, 1D convolution will be used. The convolutional layer is the key difference between a CNN and a fully connected neural network (FCNN), the number of training parameters is greatly reduced by the convolutional layer via weight sharing. Since the radar emitter signal was 1D, 1D convolution was used in this study. Compared with traditional two-dimensional convolution, 1D convolution requires fewer parameters, and there is no need for signal dimension conversion, which reduces the time cost and calculation resources necessary. The process of 1D convolution is as follows:

(5) $$y_{j}=\underset{i=C}{\sum }k_{ij}*x_{i}+b_{j}$$

where $y_{j}$ is the jth channel of the output feature map, k is the convolutional kernel, b is the bias, and C is the number of input channels. The 1D convolution is shown in Figure 1, and the height in Figure 1 is 1.

### 3.2. 1D DRSN

The difference between the DRSN [16] and general deep learning methods, like residual network (ResNet), is that the traditional ReLU is replaced by the soft thresholding function as a nonlinear activation function. A soft threshold is often used as a key step in signal denoising [17]. In traditional denoising algorithms, the signal is converted into a domain with unimportant features near zero, then these features near zero are set to 0 by the soft thresholding function. For instance, the key to the wavelet denoising algorithm is to design a filter that amplifies useful information in the signal and reduces the noise information to near zero, then the soft threshold function is used to filter the noise. However, extensive knowledge is required to design this kind of filter in reality; thus, it is often a difficult task. By integrating the soft thresholding function and the deep learning method, the DRSN can effectively improve the recognition accuracy while overcoming the difficulties associated with manually designed filters. The soft threshold function is as follows:

(6) $$y=\{\begin{array}{cc} x-\tau & x>\tau \\ 0 & -\tau \leq x\leq \tau \\ x+\tau & x<-\tau \end{array}$$

where τ is a threshold with a positive value. Different from ReLU, which sets the negative values to 0, the soft thresholding function sets the values near zero to 0, and the negative useful features are retained. The partial derivative of the soft thresholding function is as follows:

(7) $$\frac{\Delta y}{\Delta x}=\{\begin{array}{cc} 1 & x>\tau \\ 0 & -\tau \leq x\leq \tau \\ 1 & x>\tau \end{array}$$

**Figure 2:**
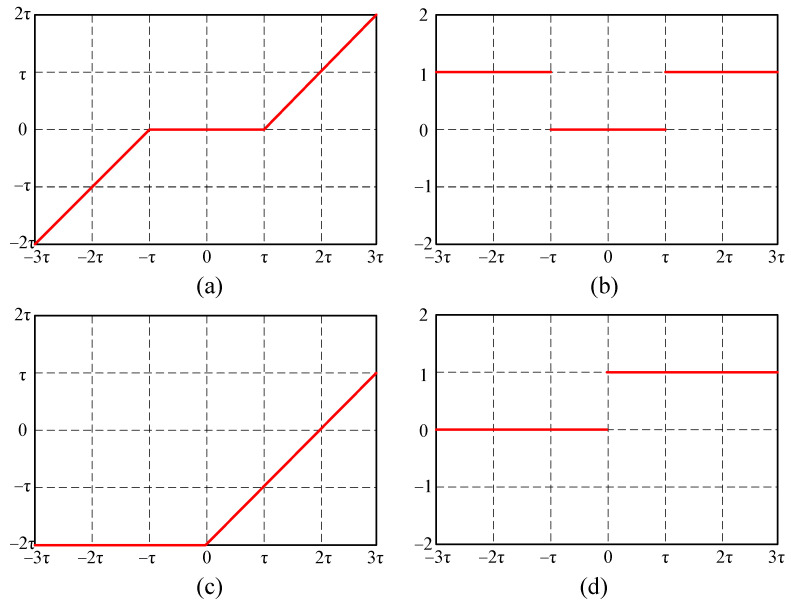
(**a**) Soft thresholding function, (**b**) derivative of the soft thresholding function, (**c**) ReLU function, and (**d**) derivative of the ReLU function.

The partial derivative of the soft thresholding function is either 1 or 0; thus, vanishing gradient and exploding gradient can be avoided. The diagrams of the soft thresholding function and ReLU function are shown in Figure 2.

**Figure 3:**
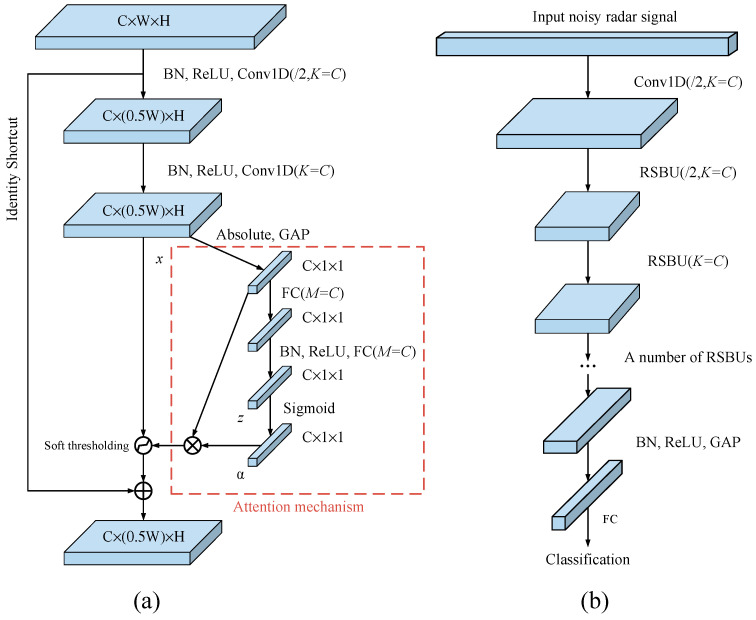
The 1D DRSN structure, (**a**) 1D residual shrinkage building unit (RSBU), and (**b**) overall framework.

The residual shrinkage units of the 1D DRSN are shown in Figure 3a, which include two batch normalization units, two ReLU activation functions, two 1D convolutional layers, one identity shortcut and one attention mechanism unit. The purpose of batch normalization is to reduce the training difficulty and to increase the training speed, and the equations are as follows:

(8) $$\mu =\frac{1}{N_{batch}}\overset{N_{batch}}{\underset{n=1}{\sum }}x_{n}$$

(9) $$\sigma^{2}=\frac{1}{N_{batch}}\overset{N_{batch}}{\underset{n=1}{\sum }}{\left(x_{n}-\mu \right)}^{2}$$

(10) $${\hat{x}}_{n}=\frac{\left(x_{n}-\mu \right)}{\sqrt{\sigma^{2}+\varepsilon }}$$

(11) $$y_{n}=\gamma {\hat{x}}_{n}+\beta$$

where $x_{n}$ and $y_{n}$ are the input features and output features of a certain batch, γ and β are two parameters that can be trained, and ε is a near-zero positive number used to prevent zero division. In batch normalization, the samples are converted to a distribution with a mean of 0 and standard deviation of 1 to reduce the training difficulty.

As one of the common activation functions in existing deep learning networks [18], the ReLU function can effectively prevent vanishing gradient and increase the training speed. The equation is as follows:

(12) y=max(x,0)

The soft threshold is determined by the attention mechanism. As shown in Figure 3a, assuming that the output after two 1D convolutional layers is x, and the output after taking the absolute value and global average pooling is:

(13) $$y_{c}=GAP\left(abs\left(x\right)\right)=\underset{i,j}{average}\left(\left|x_{i,j,c}\right|\right)$$

where y is a vector with a length of C, i, j and c are the index numbers of the width, height and channel of the feature map. The output z is obtained through two fully connected layers and is then converted to a number between 0 and 1 using the *sigmoid* function.

(14) $$a=sigmoid\left(z\right)=\frac{1}{1+e^{-z}}$$

where a is also a vector with a length of C, and the threshold is obtained after multiplication of Equations (12) and (13).

(15) $$\tau_{c}=a_{c}\cdot y_{c}$$

where $\tau_{c}$ is the threshold of channel c. The aim of Equation (14) is to emphasize that different channels may have different thresholds in the feature map with C channels. In this way, useful features can be flexibly retained, and useless features can be deleted.

Figure 3b is the overall structure of the 1D DRSN, which is similar to that of ResNet [19], the only difference is that the residual module is replaced by the 1D residual shrinkage building unit (RSBU) module, which integrates soft thresholding function and attention mechanism. Note that the DRSN consists of a series of RSBU modules and a global average pooling layer. The global average pooling layer is obtained by the mean of each channel, whose function is to reduce the number of training weights and the risk of overfitting.

(16) $$GAP^{c}=\frac{1}{N}\overset{N}{\underset{n=1}{\sum }}{x_{n}}^{c}$$

where $x^{c}$ is the output features of channel  c, and N is the number of features. The cross-entropy function is used as the error propagation function:

(17) $$L\left(\theta \right)=-\overset{k}{\underset{i=1}{\sum }}y^{i}\ln({\hat{y}}^{i})=-\overset{k}{\underset{i=1}{\sum }}y^{i}\ln(g\left(\theta ,x{)}^{i}\right)$$

where y is the data label, $\hat{y}$ is the predicted category, g(θ,x) is the output of the model, x and θ are the input and parameters of the network model, and k is the number of categories to be classified. After the cross-entropy error is calculated, the gradient descent algorithm is used for parameter optimization. To achieve fast convergence, the adaptive moment estimation (ADAM) method is used in this study, and the parameters proposed in previous studies are used [20,21].

### 3.3. Network Construction

**Figure 4:**
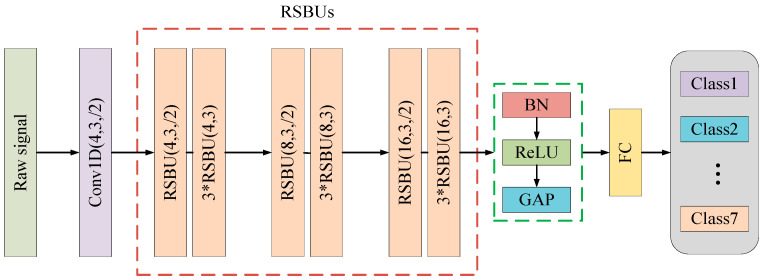
Structure of the proposed 1D DRSN.

The structure of the proposed network is shown in Figure 4. The first and second numbers in the bracket in residual shrinkage building units (RSBUs) are the number and width of the convolutional kernel, respectively. “/2” represents a step length of 2. In the RSBUs, three RSBUs were followed when the previous DRSN’s step length was 2, and there were a total of 12 RSBUs. The number of iterations was 160. To accelerate the training speed while ensuring the training accuracy, the original learning rate was set to 0.1. The learning rate decreased by 10-fold every 40 cycles until the number of iterations was 120, lastly, the learning rate of the final 40 cycles decreased by 2-fold. The number of batch samples was set to 128. To prevent overfitting of the model, we used L2 regularization, and the penalty coefficient was set to 0.0001 according to the recommendations of [22].

## 4. Results

The test platforms and parameters used in this study are shown in Table 1.

### 4.1. Datasets

Seven types of representative radar emitter signals were used, including 13-bit barker codes, frequency coding signals, frequency diversity signals, linear frequency-modulated signals (LFM), nonlinear frequency-modulated signals (NLFM), single-carrier frequency signals (CW) and barker-lfm mixed modulation signals. The SNR of the signals ranged from −8 dB to 4 dB with an interval of 2 dB, i.e., a total of seven SNRs. The sampling frequency was 512 MHz. All radar emitter signal data contained only one pulse, and the data length was 512, if the signal is less than 512 points, it shall be supplemented completely by zero-filling method. Note that the recognition of short monopulse data was more challenging. Four types of noise were generated, including Gaussian noise, Laplacian noise, Poisson noise, and Cauchy noise. Under each noise conditions:

Training set and validation set: 300 samples were generated under each SNR and signal type, a total of 7×7×300 = 14700 samples, which are randomly divided into 80% as the training set and 20% as the validation set.

Testing set: 100 samples were generated under each SNR and signal type, a total of 7×7×100 = 4900 samples.

The parameters of the signals are shown in Table 2. 

It is worth noting that the intervals of the carrier frequency and modulation parameters with different types of signals are overlapped deliberately to increase the recognition difficulty.

### 4.2. Recognition Results of Radar Signals with the Four Types of Noise

**Figure 5:**
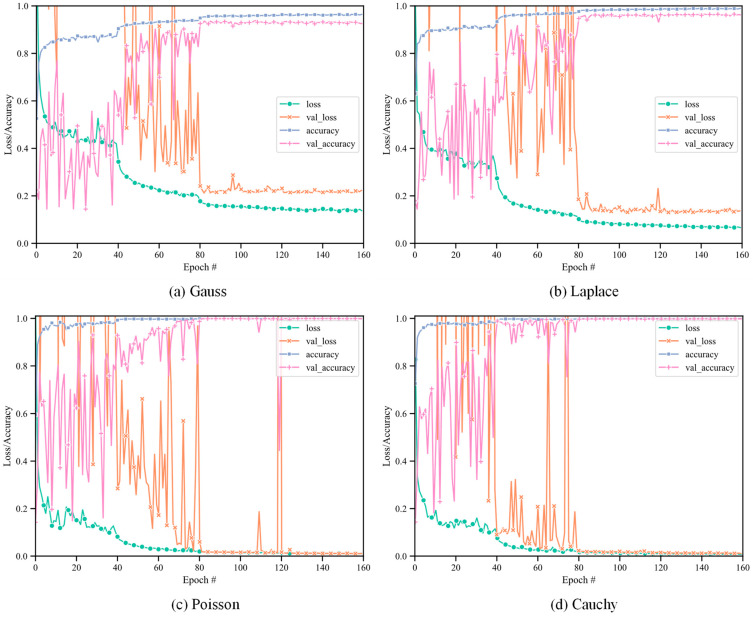
Training processing under four types of noise.

The 1D DRSN model was used for recognition of radar emitter signals with four different types of noise, i.e., Gaussian noise, Laplacian noise, Poisson noise and Cauchy noise. The average recognition rate of the training set and validation set varied with the number of iterations, as shown in Figure 5.

As Figure 5 shows, the accuracy of the training set and validation set of the four types of noise reaches a high level as the number of iterations increases, and then they were both stable after 120 iterations, indicating that the model has converged, and finally the training accuracy nearly stabilized at 96.51%, 99.00%, 99.90%, and 99.99%, respectively.

**Figure 6:**
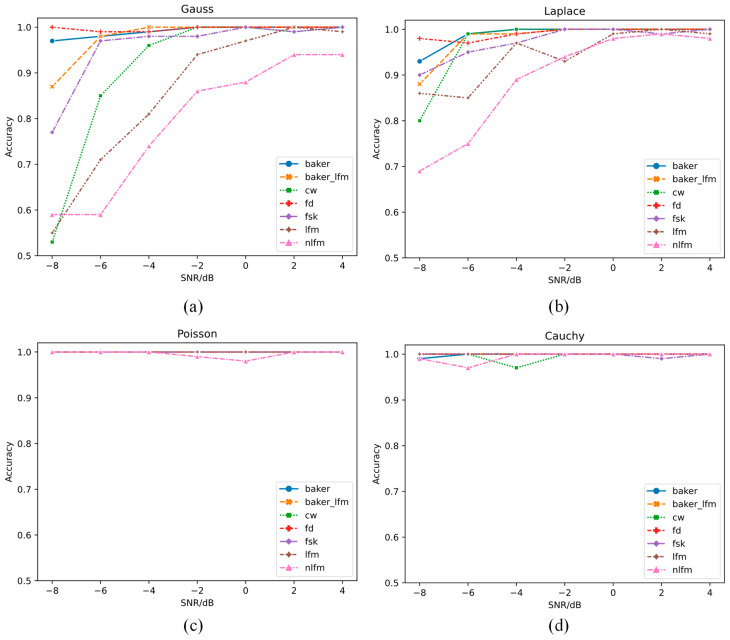
Recognition rate under (**a**) Gaussian, (**b**) Laplacian, (**c**) Poisson, and (**d**) Cauchy noise conditions.

The trained model was then evaluated using the testing set. The variation in the recognition rate with SNR under the four noise conditions is shown in Figure 6.

Figure 6a shows that under Gaussian noise, when the SNR is above −2 dB, the recognition rate reaches over 86%. Note that, except for linear frequency modulated (LFM) and nonlinear frequency modulated (NLFM) signals, the recognition rate of all signals is above 85% when the SNR is over −6 dB. The reason for the low recognition rate of LFM and NLFM signals with low SNR is that both LFM and NLFM signals are frequency modulated signals, the raw time-domain waveforms of which are very similar, and the difference in the frequency domain cannot reflected. Moreover, under the condition of strong Gaussian noise, the features of the two signals are covered, which increases the difficulty of recognition. However, the recognition accuracy of LFM and NLFM achieve 99% and 94%, respectively, when SNR is 4 dB shows that 1D DRSN is still better than the general deep learning methods. If only the frequency modulation signal needs to be recognized, the recognition rate would be improved. Under Laplacian noise, when the SNR is greater than −8 dB, the recognition rate of the 7 types of radar emitter signals is greater than 69%. Figure 6a,b show that the overall recognition rate of radar emitter signals with Laplacian noise is higher than that of the signals with Gaussian noise, indicating that the 1D DRSN model has stronger adaptability for signal recognition under non-Gaussian noise conditions. Under the condition of Poisson noise and Cauchy noise, the average recognition rate is greater than 98%, 97%, respectively, as shown in Figure 6c,d. This is because Poisson noise and Cauchy noise appear as a spike in the time-domain signal. Compared with the signal amplitude, the Poisson noise and Cauchy noise amplitude are much larger. Unlike the situation of the first two types of noise, where the time-domain waveform is submerged by the noise, the time-domain waveform is basically retained under the background of Poisson noise and Cauchy noise. Thus, the 1D DRSN model can accurately filter out the Poisson noise and Cauchy noise, and the learned features have strong discriminative ability, leading to a high average recognition rate. The recognition rate under each condition is described in detail in the Appendix A.

### 4.3. Analysis of Learned Features

**Figure 7:**
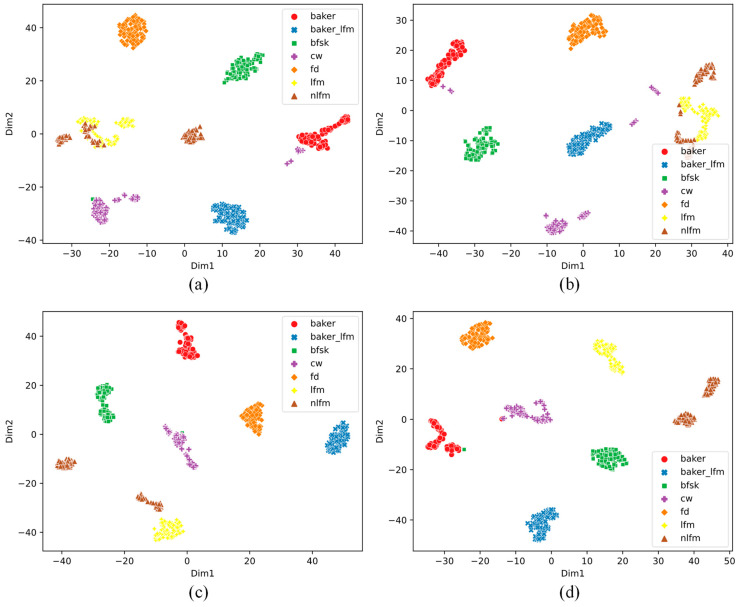
Two-dimensional visualization under(**a**) Gaussian, (**b**) Laplacian, (**c**) Poisson, and (**d**) Cauchy noise conditions.

We analyzed the features learned by the 1D DRSN model. Specifically, the test samples were input into the trained 1D DRSN model to extract the features after the global average pooling layer, and the t-distributed stochastic neighbor embedding (t-SNE) was used to reduce the dimensionality to two-dimensional space for visual analysis [23]. Although there was information loss during the dimensionality reduction process, t-SNE, as a nonlinear unsupervised dimensionality reduction method, can intuitively determine whether the learned features are distinguishable. Figure 7 shows the two-dimensional t-distribution diagrams of the radar emitter signals at 4 dB under four types of noise.

As Figure 7a,b show that, except for some of the LFM and NLFM signals being aliased, the most samples of the same type of radar emitter signal are concentrated in the same area and away from other types of samples, indicating that the features learned by the 1D DRSN have high discrimination ability. Some LFM and NLFM samples are aliased, the reason why is that the only difference in terms of amplitude is that the changing rate in the time domain. Against the noisy background, the Gaussian noise or Laplacian noise covers this unique distinguishing feature, thereby causing aliasing of LFM and NLFM samples. This also explains why the recognition rate of LFM and NLFM signals is low when the SNR is low. If the LFM and NLFM signals are regarded as frequency modulation signals, the recognition rate would be improved. In addition, Figure 7c,d show that the distance between different types of samples is large and that the same types of samples are concentrated in the same areas, indicating the high discrimination of the learned features. As Poisson noise and Cauchy noise appear as a sudden spike in the time domain, it does not affect the signal waveform, the DRSN filters out the noise well and is able to retain useful features, resulting in high signal recognition rate.

### 4.4. Comparison with Other Models

To further verify the effectiveness of the 1D DRSN, we compared the proposed method with some of the state-of-the-art deep learning networks, i.e., 1D ResNet and 1D ConvNet. The same network structure was used, as shown in Table 3.

In Table 3, RBU stands for ResNet residual module unit. Unlike RSBU, RBU uses ReLU as the activation function without the attention mechanism, so the only difference between DRSN and ResNet is the activation function, which one uses the soft thresholding function and the other uses the ReLU function. CBU represents convolution module unit, which is different from RBU and RSBU in that it has no identity connection.

**Figure 8:**
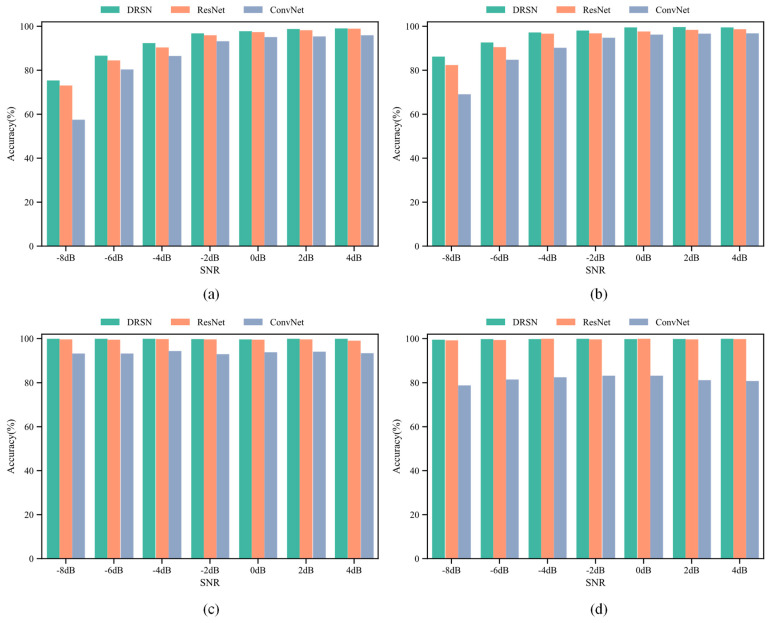
Average recognition rates of DRSN, ResNet and ConvNet under (**a**) Gaussian noise, (**b**) Laplacian noise, (**c**) Poisson noise, and (**d**) Cauchy noise.

The average recognition rates of the four models under Gaussian noise, Laplacian noise, Poisson noise and Cauchy noise are shown in Figure 8. Under all noise conditions, the average recognition rate of the 1D DRSN is higher than those of 1D ResNet and 1D ConvNet, indicating that the 1D DRSN model has a better performance, which is because 1D DRSN uses a soft thresholding activation function to retain useful features of the signal while eliminate the useless features of noise.

Table 4, Table 5, Table 6 and Table 7 show the average recognition accuracies of DRSN, ResNet and ConvNet under four kinds of noise conditions on the test set, which indicated that the DRSN model has a better performance for radar emitter signal recognition under noisy condition.

Table 8 shows the number of parameters and training time per epoch for DRSN, ResNet and ConvNet models, respectively. It can be seen that the DRSN improves recognition accuracy without significantly increasing the consuming of time and computing resources.

### 4.5. Comparison with Different Sampling Frequencies

**Figure 9:**
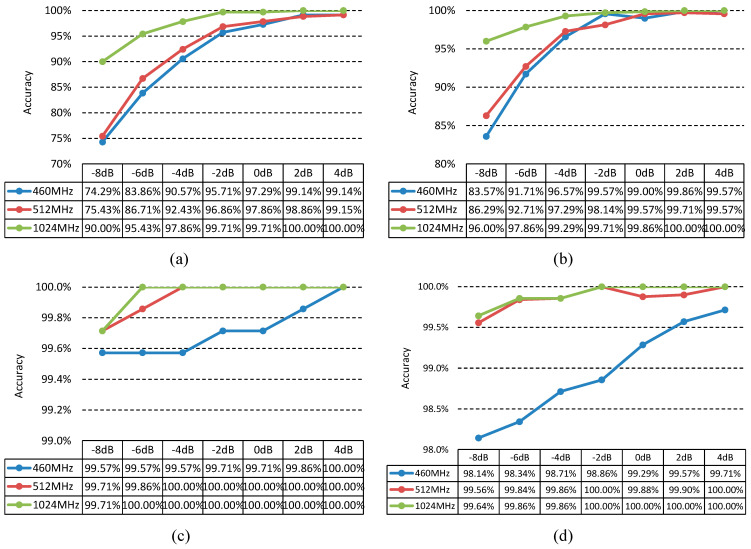
Average recognition rate with different sampling frequencies under (**a**) Gaussian noise, (**b**) Laplacian noise, (**c**) Poisson noise, and (**d**) Cauchy noise.

Under the condition of Nyquist sampling theorem, the data sets with sampling frequencies of 460 MHz, 512 MHz and 1024 MHz were used to compare the effects of different sampling frequencies on the performance of 1D DRSN. The results are shown in Figure 9. 

As can be seen from Figure 9, the greater sampling frequency, the higher recognition rate, in particular, the lower SNR, the more obvious improvement of accuracy. This is because the larger the sampling frequency, the more information obtained, and the more effective discriminant features the 1D DRSN can learn, but it also consumes more time and computing resources.

The average accuracy and training time at different sampling frequencies are shown in Table 9.

## 5. Comparison between the Soft Thresholding Function and ReLU Function

In order to elaborate the mechanism of eliminating redundant features of soft thresholding function, the soft thresholding function and ReLU function are compared and analyzed in this section. Both of them can set the features of part of the interval to 0 and delete useless features. As mentioned earlier, the gradient of the soft thresholding function and ReLU is either 1 or 0, both of which are conducive to error back propagation. Compared with ReLU, the advantage of the soft thresholding function is that the threshold can be set flexibly, thereby more accurately deleting useless feature intervals while retaining useful features. The soft thresholding function is expressed as follows:

(18) y=sign(x)⋅max(abs(x)−τ,0)

**Figure 10:**
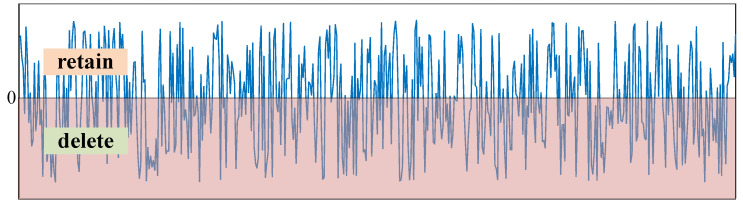
ReLU function when *b* = 0.

Considering the bias when training 1D DRSN model, when the bias is b=0:

The ReLU function sets all negative features to 0, as shown in Figure 10, the red shaded part indicates that the feature of the part is set to 0.

**Figure 11:**
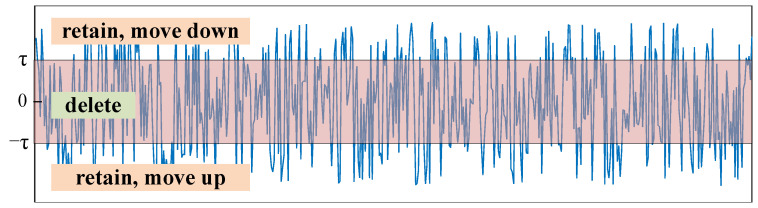
The soft thresholding function when b=0.

Similarly, the soft thresholding function is shown as Figure 11 when the bias is b=0, the features in the interval [−τ,τ] are deleted, and the rest are retained and move to the 0 by τ.

**Figure 12:**
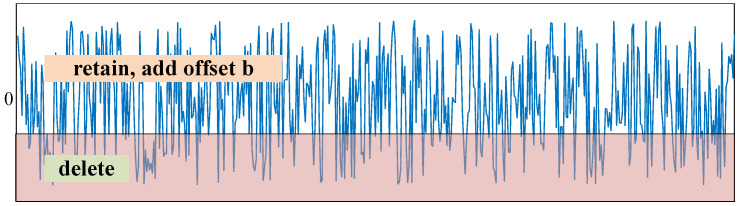
ReLU function when b>0.

**Figure 13:**
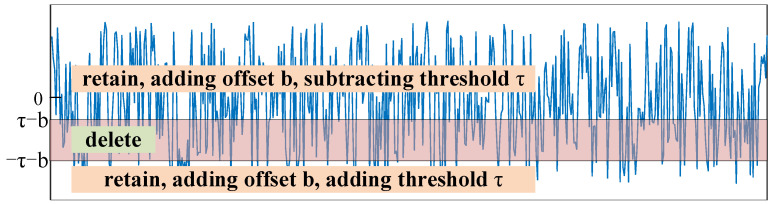
The soft thresholding function when *b* > 0.

**Figure 14:**
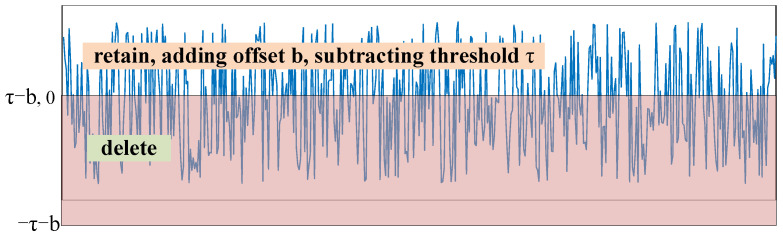
The soft threshold function when *b = τ.*

When the bias b≠0, here, we analyze only the situation when *b* > 0 in consideration of the length of the paper. When b>0:

The ReLU function becomes y=max(x+b,0), and the feature values are shifted up by b; then the negative features are set to 0, as shown Figure 12:

The soft thresholding function becomes y=sign(x+b)⋅max(abs(x+b)−τ,0). The feature values are first shifted up by b, and then feature values in the threshold interval are set to 0, as shown Figure 13:

Next, we analyze the reason why soft thresholding function is better than ReLU function as the activation function. The explanation is that the former can achieve the same function as ReLU when given proper values of b and τ, but the converse is not. In fact, in 1D DRSN, the bias b and threshold τ are both trainable parameters. Due to the data of radar signals are finite, the feature values are distributed in a certain interval. When

(19) $$b=\tau \;\mathrm{and}\;b>-\frac{1}{2}\min(x)$$

the soft thresholding function is equivalent to the ReLU function with a bias of 0, as shown Figure 14:

Similarly, the soft thresholding function is equivalent to the ReLU function with a non-zero bias when the following equation is satisfied:

(20) −τ−b<min(x)

Instead, no matter how ReLU + bias combined, it cannot achieve the same functions as the soft thresholding function. Therefore, the soft thresholding function has a more flexible deletion interval than ReLU, which makes it more flexible and reliable when removing useless features caused by noise, moreover, the 1D DRSN uses an attention mechanism to adaptively set an appropriate threshold for each sample. Thus, the 1D DRSN is suitable for situations where the noise level of each sample is different. As a result, the 1D DRSN achieves a better result in the recognition of noisy radar emitter signals.

## 6. Conclusions

In this study, we proposed a radar emitter signal recognition method based on 1D DRSN and experimentally showed the following advantages of this method: (i) by using the soft thresholding function with attention mechanism, a high recognition rate can be achieved for different types of strong noise; and (ii) no professional knowledge of signal processing or dimension conversion of data is needed, and the 1D DRSN can automatically learn the features of the signal directly from the 1D data.

The 1D DRSN outperformed traditional deep learning methods, improving the average recognition rate by 6.18% and 1.00% compared with the 1D ConvNet and ResNet, respectively. It shows that 1D DRSN can effectively improve the recognition rate of noisy radar emitter signals.

Lastly, the mechanism of the soft thresholding function was analyzed, and the reason why the soft thresholding function outperforms ReLU was discussed. The result suggested that the soft thresholding function is suitable for the recognition of noisy highly noised radar emitter signals.

## Figures and Tables

**Table 1:** Experiment platform parameters.

Project	Parameter
CPU	i7-10700
GPU	RTX 2060
RAM	16G
Simulation Software	MATLAB2020a, Python3.7, Tensorflow2.2

**Table 2:** Specific parameters of seven types of radar emitter signals.

Signal Type	Carrier Frequency	Parameter
Barker	10~30 MHz	13-bit Barker code width of each symbol is 1/13 us
Barker-lfm	10~30 MHz	Frequency bandwidth: 100 MHz to 200 MHz 13-bit Barker code width of each symbol is 1/13 us
Frequency-coding	10~20 MHz 100~200 MHz	13-bit random code width of each symbol is 1/13 us
Frequency diversity	10~20 MHz 50~60 MHz 90~100 MHz	None
LFM	20~30 MHz	Frequency bandwidth: 50 MHz to 200 MHz 1/2 up frequency modulation 1/2 down frequency modulation
NLFM	20~30 MHz	Frequency bandwidth: 50 MHz to 200 MHz Modulation: Quadratic 1/2 up frequency modulation 1/2 down frequency modulation
CW	10~30 MHz	None

**Table 3:** Structural parameters of the three models.

Number of Blocks	Output Size	DRSN	ResNet	ConvNet
1	1 × 512 × 1	Input	Input	Input
1	4 × 256 × 1	Conv (4, 3, /2)	Conv (4, 3, /2)	Conv (4, 3, /2)
1	4 × 128 × 1	RSBU (4, 3, /2)	RBU (4, 3, /2)	CBU (4, 3, /2)
3	4 × 128 × 1	RSBU (4, 3)	RBU (4, 3)	CBU (4, 3)
1	8 × 64 × 1	RSBU (8, 3, /2)	RBU (8, 3, /2)	CBU (8, 3, /2)
3	8 × 64 × 1	RSBU (8, 3)	RBU (8, 3)	CBU (8, 3)
1	16 × 32 × 1	RSBU (16, 3, /2)	RBU (16, 3, /2)	CBU (16, 3, /2)
3	16 × 32 × 1	RSBU (16, 3)	RBU (16, 3)	CBU (16, 3)
1	16	BN, ReLU, GAP	BN, ReLU, GAP	BN, ReLU, GAP
1	7	FC	FC	FC

**Table 4:** Average accuracies of the DRSN, ResNet and ConvNet with Gaussian noise (%).

Method	Test Accuracy
DRSN	92.25
ResNet	91.26
ConvNet	86.34

**Table 5:** Average accuracies of the DRSN, ResNet and ConvNet with Laplacian noise (%).

Method	Test Accuracy
DRSN	96.18
ResNet	94.49
ConvNet	89.86

**Table 6:** Average accuracies of the DRSN, ResNet and ConvNet with Poisson noise (%).

Method	Test Accuracy
DRSN	99.94
ResNet	99.61
ConvNet	93.63

**Table 7:** Average accuracies of the DRSN, ResNet and ConvNet with Cauchy noise (%).

Method	Test Accuracy
DRSN	99.88
ResNet	99.71
ConvNet	81.67

**Table 8:** The number of parameters and training time per epoch for DRSN, ResNet and ConvNet.

Model	DRSN	ResNet	ConvNet
Quantity of parameters	12,215	8855	8855
Time per epoch	2.56 s	1.57 s	1.55 s

**Table 9:** The average accuracy (%) and training time (s) of DRSN at different sampling frequencies.

Frequency	460 MHz	512 MHz	1024 MHz
Average accuracy	96.45	97.11	99.05
Time	405.63	409.50	417.22

## Data Availability

Not applicable.

## Appendix A

The recognition results in detail for Figure 6a–d are shown in Table A1, Table A2, Table A3 and Table A4, respectively.

**Table A1:** Recognition results of one-dimensional DRSN under Gaussian noise.

	SNR	−8 dB	−6 dB	−4 dB	−2 dB	0 dB	2 dB	4 dB
Signal								
baker		0.97	0.98	0.99	1.00	1.00	1.00	1.00
baker_lfm		0.87	0.98	1.00	1.00	1.00	1.00	1.00
frequency -coding		0.77	0.97	0.98	0.98	1.00	0.99	1.00
frequency diversity		1.00	0.99	0.99	1.00	1.00	1.00	1.00
lfm		0.55	0.71	0.81	0.94	0.97	1.00	0.99
nlfm		0.59	0.59	0.74	0.86	0.88	0.94	0.94
cw		0.53	0.85	0.95	1.00	1.00	0.99	1.00

**Table A2:** Recognition results of one-dimensional DRSN under Laplacian noise.

	SNR	−8 dB	−6 dB	−4 dB	−2 dB	0 dB	2 dB	4 dB
Signal								
baker		0.93	0.99	1.00	1.00	1.00	1.00	1.00
baker_lfm		0.88	0.99	0.99	1.00	1.00	1.00	1.00
frequency-coding		0.89	0.94	0.97	1.00	1.00	0.99	1.00
frequency diversity		0.98	0.97	0.99	1.00	1.00	1.00	1.00
lfm		0.86	0.85	0.97	0.93	0.99	1.00	0.99
nlfm		0.68	0.75	0.89	0.93	0.98	0.99	0.98
cw		0.80	0.99	1.00	1.00	1.00	1.00	1.00

**Table A3:** Recognition results of one-dimensional DRSN under Poisson noise.

	SNR	−8 dB	−6 dB	−4 dB	−2 dB	0 dB	2 dB	4 dB
Signal								
baker		1.00	1.00	1.00	1.00	1.00	1.00	1.00
baker_lfm		1.00	1.00	1.00	1.00	1.00	1.00	1.00
frequency coding		1.00	1.00	1.00	1.00	1.00	1.00	1.00
frequency diversity		1.00	1.00	1.00	1.00	1.00	1.00	1.00
lfm		1.00	1.00	1.00	1.00	1.00	1.00	1.00
nlfm		1.00	1.00	1.00	0.99	0.98	1.00	1.00
cw		1.00	1.00	1.00	1.00	1.00	1.00	1.00

**Table A4:** Recognition results of one-dimensional DRSN under Cauchy noise.

	SNR	−8 dB	−6 dB	−4 dB	−2 dB	0 dB	2 dB	4 dB
Signal								
baker		0.99	1.00	1.00	1.00	1.00	1.00	1.00
baker_lfm		1.00	1.00	1.00	1.00	1.00	1.00	1.00
frequency coding		1.00	1.00	1.00	1.00	1.00	0.99	1.00
frequency diversity		1.00	1.00	1.00	1.00	1.00	1.00	1.00
lfm		1.00	1.00	1.00	1.00	1.00	1.00	1.00
nlfm		0.99	0.97	1.00	0.99	0.98	1.00	1.00
cw		1.00	1.00	0.97	1.00	1.00	1.00	1.00
